# Primary duodenal papilla lymphoma producing obstructive jaundice: a case report

**DOI:** 10.1186/s12893-022-01558-3

**Published:** 2022-03-23

**Authors:** Jicai Wang, Jiantao Han, Hanbing Xu, Sheng Tai, Xingwang Xie

**Affiliations:** 1 Department of Hepatopancreatobiliary, Thyroid and Vascular Surgery, Wuhan Third Hospital (Tongren Hospital of Wuhan University), No. 241 Pengliuyang Road, Wuchang, Wuhan, 430060 Hubei China; 2 Department of Hepatic Surgery, The Second Affiliated Hospital of Harbin Medical University, No. 246 XueFu Avenue, Harbin, 150086 Heilongjiang China

**Keywords:** Lymphoma, Duodenum, Duodenal papilla, Jaundice

## Abstract

**Background:**

Obstructive jaundice caused by primary duodenal lymphoma is a rare disease.

**Case presentation:**

We reported a 59-year-old man who underwent endoscopic ultrasonography for obstructive jaundice and found a duodenal papilla tumor. Light microscopy revealed a non-Hodgkin's lymphoma. Immunohistochemical staining showed that the tumor was aggressive B-cell lymphoma. We carried out molecular targeted therapy combined with CHOP regimen chemotherapy.

**Conclusion:**

Surgery plays an important role in resolving obstructive jaundice when accurate histological diagnosis cannot be made. After diagnosis, chemotherapy should play a central role in treatment.

## Background

Isaacson and Wright first proposed the concept of mucosa associated lymphoid tissue lymphoma in 1983. Under the stimulation of long-term chronic inflammation, extranodal organs may form mucosa-associated lymphoid tissue lymphoma. The most common site of occurrence is digestive tract, followed by lung, salivary gland, head and neck, eye accessories, skin, thyroid, breast and so on [1, 2]. In gastrointestinal lymphoma, while the stomach accounts for most cases, duodeno-papillary lymphoma is extremely rare [3–5]. We reported a case of primary duodenal papilla invasive B-cell lymphoma with obstructive jaundice as the first symptom.

## Case presentation

**Fig. 1:**
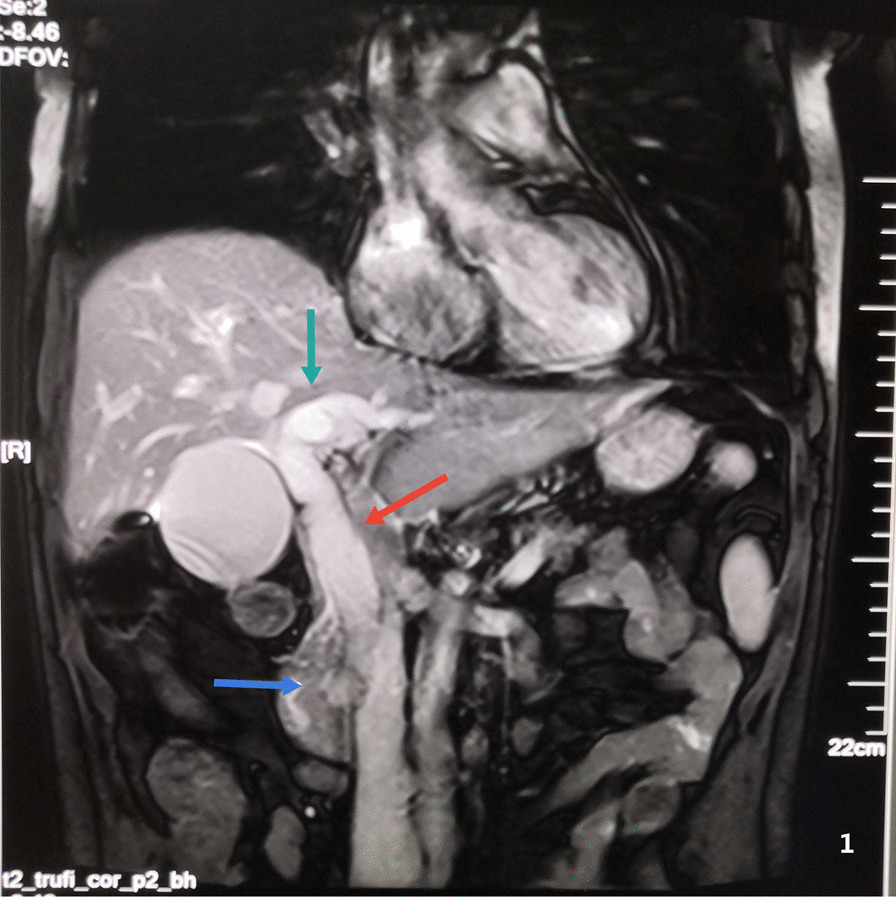
Abdominal MRI. Blue arrow: neoplastic lesions of the duodenal papilla. Green and red arrows: severe dilatation of intrahepatic and extrahepatic bile ducts

**Fig. 2:**
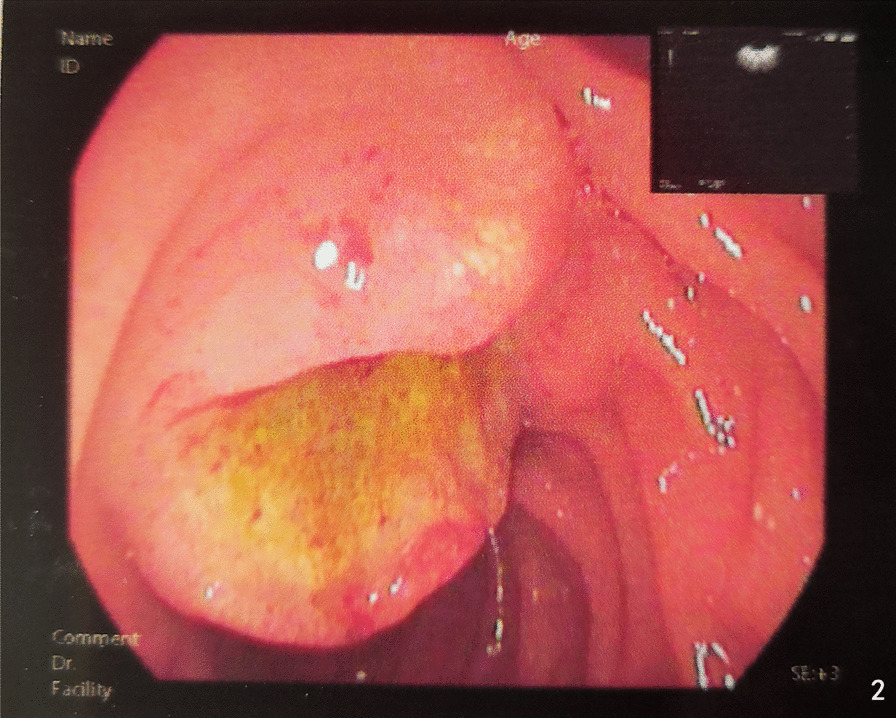
Ultrasonic duodenoscopy: duodenal papilla occupying space with cholangiopancreatic duct dilatation

**Fig. 3:**
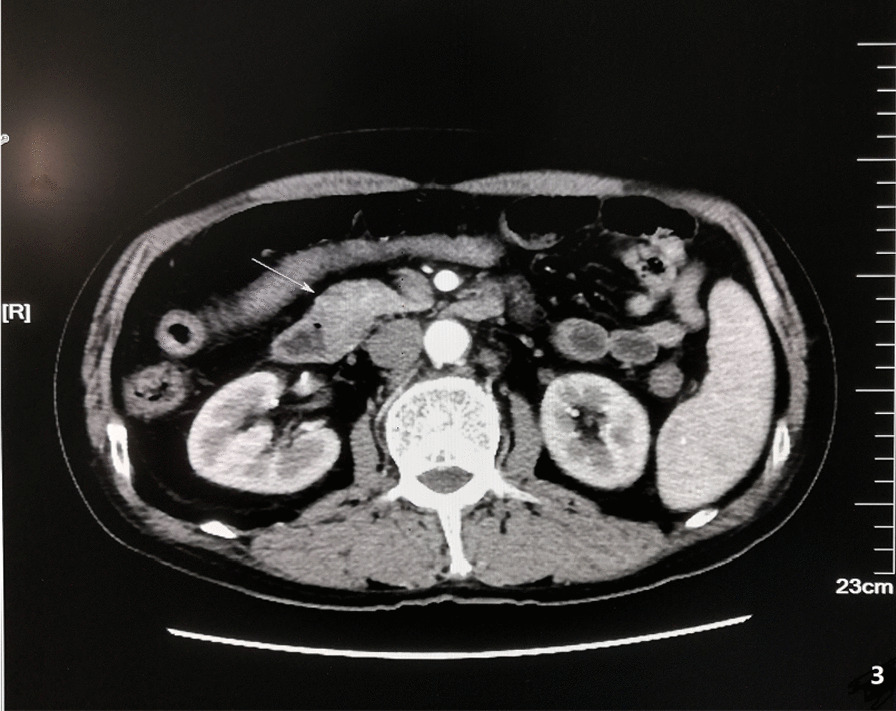
Abdominal enhancement CT: duodenal papilla space-occupying lesions, intrahepatic and extrahepatic bile duct dilatation

**Fig. 4:**
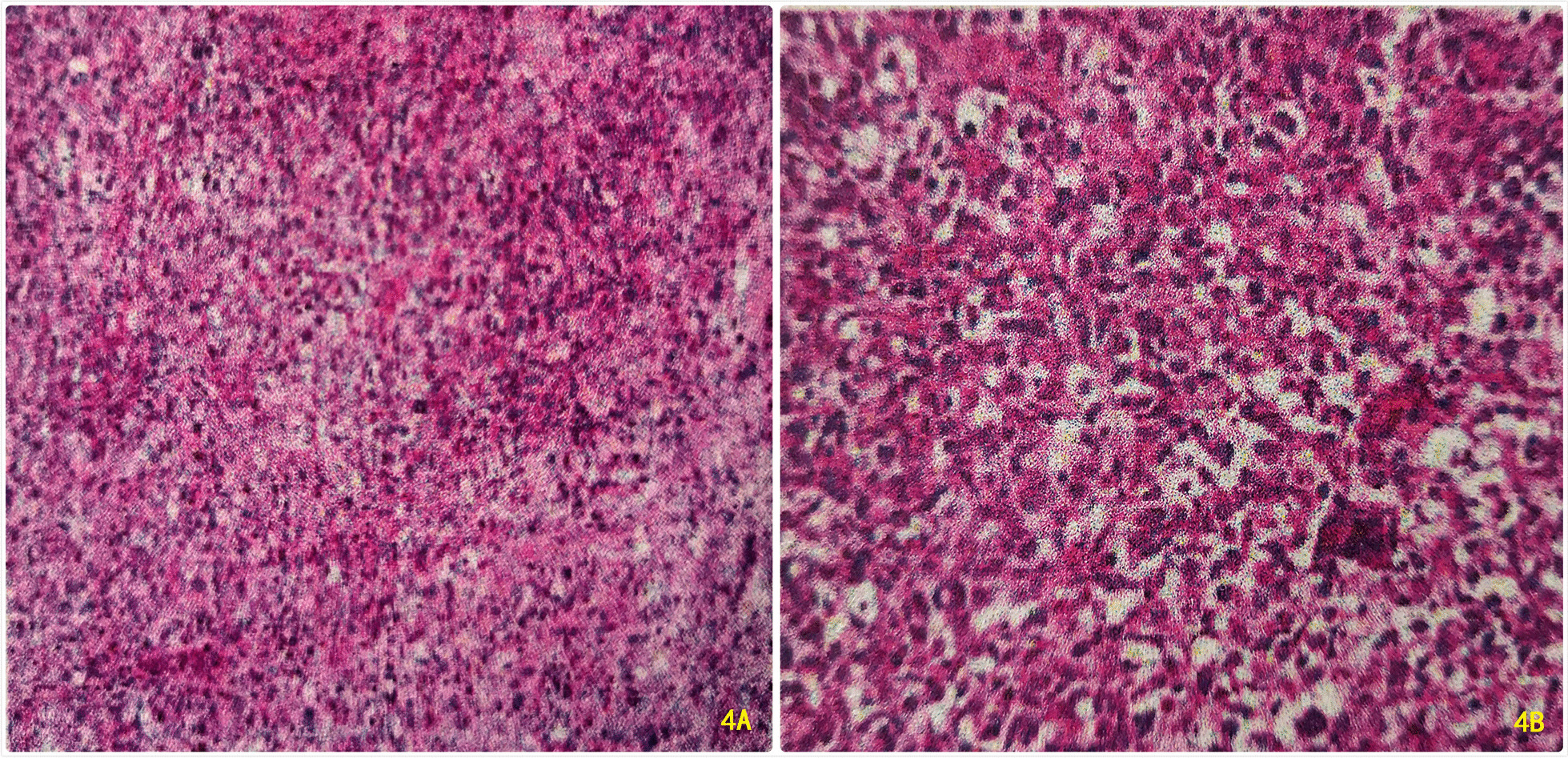
**A**, **B** Pathology of ultrasonic gastroscope biopsy: duodenal papilla non-Hodgkin’s lymphoma, invasive B-cell lymphoma, LCA ( +), CD34 (vascular +), CD20 ( +). **A**: (HE × 40); **B**: (HE × 100)

A 59 year old male was admitted to the Department of Gastroenterology of our hospital on June 16, 2020 due to jaundice of the skin and sclera for more than 15 days and progressive weight loss about 5 kg. He had a history of brain stem infarction with left lower limb weakness and occasionally chokes on drinking water. He denied a history of hepatitis, tuberculosis or other infectious diseases. Physical examination showed that he had chronic disease appearance with jaundice of the skin and sclera, but no obvious enlargement of superficial lymph nodes and no obvious positive signs in the abdomen. Laboratory data on the day of admission were as follows: hemoglobin 101 g/L, glutamic-pyruvic transaminase 158 U/L, glutamic-oxaloacetic transaminase 150 U/L, total bilirubin 217.3 umo/L, direct bilirubin 152.4 umo/L, indirect bilirubin 64.9 umol/L, hypersensitive C-reactive protein 5.8 mg/L, procalcitonin 0.11 ng/ml, carcinoembryonic antigen (CEA) 6.19 μg/L, carbohydrate antigen 125 (CA125) 117.1 kU/L. On June 17 abdominal ultrasonography showed gallbladder enlargement, cholecystitis, cholestasis, and dilatation of intrahepatic and extrahepatic bile ducts. On June 18 magnetic resonance cholangiopancreatography (MRCP) and abdominal magnetic resonance (MRI) (Fig. 1) considered neoplastic lesions of the duodenal papilla, severe dilatation of intrahepatic and extrahepatic bile ducts, bile stasis in common bile duct, large gallbladder. On June 19 ultrasound gastroscopy (Fig. 2) revealed that a neoplasms of approximately 2.5 × 2.5 cm in size could be seen at the duodenal papilla, with ulceration on the surface, covered with yellow and white slough, and contact bleeding. Three biopsies were taken. The patient was transferred to our department from the Department of Gastroenterology on June 20. The results of abdominal enhanced computed tomography (CT) (Fig. 3) on June 23 were similar to MRI. On the same day cholangiograms of percutaneous transhepatic biliary drainage (PTBD) was performed for jaundice reduction. On June 29 we prepped the patient for pancreaticoduodenectomy after discussion and obtaining informed consent from the patient and his family. However, histopathological examination on June 30 (Fig. 4A and B) showed severe chronic active inflammation with ulcers, infiltrated by large number of atypical lymphocytes. Mitotic figures were visible, and the cytoplasm of tumor cells was empty and bright. Immunohistochemical results were as follows: LCA ( +), CD34 (vascular +), Ki67 (Li80%), CD20 ( +), LCA ( +), CD34 (vascular +). Aggressive Non-Hodgkin’s B-cell lymphoma was diagnosed on the basis of this results. The surgical plan was cancelled after a consultation with a haematology, chemotherapy was indicated.

## Discussion and conclusions

Lymphoma is a type of malignant tumor originating in the lymphohematopoietic system. Due to the characteristics of the systemic distribution of the lymphatic system, lymphoma can invade any tissue and organ in the whole body. The main clinical manifestations are painless lymphadenopathy and hepatosplenomegaly [6]. Based on the tumor cell origin, lymphoma can be divided into Hodgkin lymphoma and non-Hodgkin lymphoma. Hodgkin lymphoma can be divided into classical type and nodular lymphocyte-based type according to the histological subtype. The former can also be divided into four histological subtypes: nodular sclerosis type, lymphocyte-rich type, mixed cell type and lymphocyte-depleted type. The clinical incidence of non-Hodgkin's lymphoma is much higher than that of Hodgkin's lymphoma. Non-Hodgkin's lymphoma can be classified as B cell, T cell, or natural killer cell lymphoma depending on the lymphocyte origin [7].

Among all gastrointestinal malignancies, small intestinal tumors account for only 1–2% of them [8]. Lymphoma only accounts for 4–11% of small intestinal malignancies, most of which occur in the ileum, followed by jejunum and duodenum [9]. Duodenal lymphoma accounts for only 12% of all duodenal malignancies [10]. Obstructive jaundice is often caused by benign diseases such as hepatolithiasis and cholangitis, and also by malignant diseases such as cholangiocarcinoma, periampullary cancer, liver cancer and pancreatic cancer. In this case, malignant obstructive jaundice caused by duodenal papillary B-cell lymphoma is extremely rare [11, 12].

In combination with this case, the author has the following experiences: (1) duodenal lymphoma usually has no specific clinical symptoms in the early stage, mainly non-specific manifestations such as abdominal distension, pain and discomfort, which can cause obstructive manifestations when the tumor obstructs the duodenum. The B-cell lymphoma in this patient grew impartially at the opening of the large duodenal papilla and obstructed the pancreaticobiliary duct, affecting biliopancreatic secretion, resulting in severe dilatation of the pancreaticobiliary duct. The patient was treated with obstructive jaundice as the first symptom. (2) The patient showed no other obvious positive signs except jaundice of the skin and sclera. It is very easy to cause misdiagnosis and missed diagnosis in the clinic due to the atypical clinical symptoms and signs, combined with the lack of understanding of the disease. (3) In this case, bilirubin and aminotransferase were significantly increased in this patient, abdominal color Doppler ultrasound suggested dilatation of intrahepatic and extrahepatic bile ducts, abdominal enhanced MRI, gastroscopy with endoscopic ultrasound and abdominal enhanced CT all indicated duodenal papillary occupying lesions, and lung CT showed no obvious abnormalities. These auxiliary investigations are helpful in determining the definitive diagnosis as well as determine stage the disease, including local invasion of major vessels and distant metastasis. (4) In the last half month, the patient had a significant weight loss (about 5 kg) and intermittent fever. All the above results and clinical manifestations seemed to indicate duodenal papillary tumor combined with biliary tract infection. Influenced by the past experience, the next treatment should be PTBD to reduce jaundice, control infection, strengthen nutrition, improve anemia and other preoperative preparations, preoperative discussion and consent, and then pancreatoduodenectomy. In fact, we were also prepared in this way until the day before the surgery that histopathological results of biopsy under ultrasound gastroscopy were duodenal papillary non-Hodgkin lymphoma, and aggressive B-cell lymphoma. Afterwards, we invited the hematology department for consultation, changed the treatment regimen, suspended the operation, and applied rituximab targeted therapy combined with CHOP regimen chemotherapy. (5) Frequent fever in this patient's course easily confuses neoplastic fever caused by lymphoma with infectious fever caused by biliary tract infection. In addition, upon review of the histopathology results, we carefully examined the patient again, but there were not palpable superficial lymph nodes. Subsequently, cervical lymph nodes and inguinal lymph nodes color Doppler ultrasound showed lymph node enlargement.

Obstructive jaundice caused by duodenal papillary lymphoma is extremely rare. Establishment of histology, immunohistochemistry and molecular detection is essential for diagnosis and treatment. Different histological subtypes have different treatment and prognosis. Surgery plays an important role in resolving obstructive jaundice when accurate histological diagnosis cannot be made. After diagnosis, chemotherapy should play a central role in treatment.

## Data Availability

The datasets used and analyzed during the current study are available from the corresponding author on reasonable request.

## Abbreviations

CEA: Carcinoembryonic antigen
CA125: Carbohydrate antigen 125
MRCP: Magnetic resonance cholangiopancreatography
MRI: Magnetic resonance
CT: Computed tomography
PTCD: Percutaneous transhepatic cholangiodrainage
